# Body size throughout the life-course and incident benign prostatic hyperplasia-related outcomes and nocturia

**DOI:** 10.1186/s12894-021-00816-5

**Published:** 2021-03-27

**Authors:** Saira Khan, K. Y. Wolin, R. Pakpahan, R. L. Grubb, G. A. Colditz, L. Ragard, J. Mabie, B. N. Breyer, G. L. Andriole, S. Sutcliffe

**Affiliations:** 1 Epidemiology Program, College of Health Sciences, University of Delaware, 100 Discovery Blvd., 7th floor, Newark, DE 19713 USA; 2 Coeus Health, 222 W Merchandise Mart Plaza, Chicago, IL 60654 USA; 3 Division of Public Health Sciences, Department of Surgery, Washington University in St. Louis School of Medicine, 660 S. Euclid Ave., Campus Box 8100, St. Louis, MO 63110 USA; 4 Department of Urology, Medical University of South Carolina, 135 Rutledge Ave, Charleston, SC 29425 USA; 5 Westat, 1600 Research Blvd, Rockville, MD 20850 USA; 6 Information Management Services, Inc., 1455 Research Blvd, Suite 315 , Rockville, MD 20850 USA; 7 Departments of Urology and Epidemiology and Biostatistics, University of California - San Francisco, 400 Parnassus Ave # 610, San Francisco, CA 94143 USA; 8 Division of Urologic Surgery, Department of Surgery, Washington University in St. Louis School of Medicine, 4921 Parkway Place, St. Louis, MO 63110 USA

**Keywords:** Benign Prostatic Hyperplasia (BPH), Body size, Obesity, Nocturia, Prostate volume, PLCO

## Abstract

**Background:**

Existing evidence suggests that there is an association between body size and *prevalent* Benign Prostatic Hyperplasia (BPH)-related outcomes and nocturia. However, there is limited evidence on the association between body size throughout the life-course and *incident* BPH-related outcomes.

**Methods:**

Our study population consisted of men without histories of prostate cancer, BPH-related outcomes, or nocturia in the intervention arm of the Prostate, Lung, Colorectal, and Ovarian Cancer Screening Trial (PLCO) (n = 4710). Associations for body size in early- (age 20), mid- (age 50) and late-life (age ≥ 55, mean age 60.7 years) and weight change with incident BPH-related outcomes (including self-reported nocturia and physician diagnosis of BPH, digital rectal examination-estimated prostate volume ≥ 30 cc, and prostate-specific antigen [PSA] concentration > 1.4 ng/mL) were examined using Poisson regression with robust variance estimation.

**Results:**

Men who were obese in late-life were 25% more likely to report nocturia (Relative Risk (RR): 1.25, 95% Confidence Interval (CI): 1.11–1.40; p-trend_for continuous BMI_ < 0.0001) and men who were either overweight or obese in late-life were more likely to report a prostate volume ≥ 30 cc (RR_overweight_: 1.13, 95% CI 1.07–1.21; RR_obese_: 1.10, 95% CI 1.02–1.19; p-trend_for continuous BMI_ = 0.017) as compared to normal weight men. Obesity at ages 20 and 50 was similarly associated with both nocturia and prostate volume ≥ 30 cc. Considering trajectories of body size, men who were normal weight at age 20 and became overweight or obese by later-life had increased risks of nocturia (RR_normal to overweight_: 1.09, 95% CI 0.98–1.22; RR_normal to obese_: 1.28, 95% CI 1.10–1.47) and a prostate volume ≥ 30 cc (RR_normal to overweight_: 1.12, 95% CI 1.05–1.20). Too few men were obese early in life to examine the independent effect of early-life body size. Later-life body size modified the association between physical activity and nocturia.

**Conclusions:**

We found that later-life body size, independent of early-life body size, was associated with adverse BPH outcomes, suggesting that interventions to reduce body size even late in life can potentially reduce the burden of BPH-related outcomes and nocturia.

**Supplementary Information:**

The online version contains supplementary material available at 10.1186/s12894-021-00816-5.

## Introduction

Benign prostatic hyperplasia (BPH)-related outcomes and lower urinary tract symptoms (LUTS) are extremely common among middle- and older-aged American men. By the time men reach their sixties approximately half are believed to have prevalent BPH/LUTS [1–3]. LUTS contribute to considerable bother and decreased quality of life among older men [4], and may lead to severe complications [5] as well as high healthcare costs [6].

Body size and obesity are risk factors for many inextricably linked conditions, including many sex-hormone related diseases, through a complex set of interrelated mechanisms. Specifically, recent interest in the role of body size in BPH/LUTS prevention has grown [7–9]. Greater body size, particularly obesity, may promote BPH/LUTS through several possible mechanisms. First, obesity increases the ratio of estrogens to testosterone and its metabolites, thus possibly exaggerating the natural increase that occurs with aging in men. This increase in the estrogen to testosterone ratio has been shown to contribute to BPH in dogs [10] and proposed to contribute to BPH/LUTS development in men. Second, obese individuals have higher levels of systemic inflammation and oxidative stress. Both systemic inflammation and oxidative stress have been proposed to promote unregulated prostate growth based on the frequent observation of inflammation in BPH tissue, and the observed correlation between the extent and severity of inflammation and the degree of prostate enlargement [11]. Finally, excess body weight may exert increased intra-abdominal pressure on the bladder and increased intravesical pressure, causing or exacerbating LUTS [12].

Consistent with these mechanisms, recent epidemiological reviews have observed positive associations between body mass index (BMI) and the presence of BPH and LUTS [13]. However, most of these studies did not exclude men with LUTS at baseline from their analyses. This approach is concerning because LUTS may cause men to alter their behaviors (e.g., reduce physical activity), which in turn may influence their BMI and contribute to misleading positive associations. Interestingly, fewer studies have investigated *incident* BPH-related outcomes in men free of LUTS at baseline to avoid the concern of reverse causation. Findings from this smaller number of incident (rather than prevalent) studies have been variable [14–23].

As the underlying pathology of BPH/LUTS is believed to begin in men as early as their twenties or thirties [24], early-life body size may also potentially contribute to BPH/LUTS risk. However, only a few studies, to our knowledge, have investigated body size earlier in life or across the life-course in relation to BPH/LUTS [10, 17, 23, 25–27], and most were limited to prevalent disease. Therefore, many of these early-life studies are still subject to the same methodologic concerns as described above for studies of later-life body size. Accurately assessing the relationship between earlier-life BMI and BPH/LUTS development is important to inform the necessary timing of future preventive interventions, i.e., whether body size reduction must be addressed earlier in life or whether a reduction in body size even later in life may still reduce BPH/LUTS risk.

Finally, results from investigations of other disease outcomes have suggested that obesity and physical inactivity may act synergistically on disease processes [28]. Our previous analyses in this cohort found significant reductions in the incidence of some BPH-related outcomes for men who were physically active [29]. However, few studies have examined whether the benefits of physical activity are the same for obese and lean men.

We investigated the relationship between body size and risk of BPH-related outcomes and nocturia in the large Prostate, Lung, Colorectal, and Ovarian Cancer Screening Trial (PLCO). Importantly, this is one of the first studies to examine early-, mid-, and late-life body size, as well changes in body size throughout the life-course, with *incident* BPH/LUTS.

## Methods

### Study population

PLCO is a large, ongoing clinical trial designed to investigate the effects of prostate, lung, colorectal, and ovarian cancer screening on cancer-specific mortality [30]. From 1993 to 2001, men were recruited into PLCO at 10 screening centers across the U.S. Each site obtained IRB approval and consented participants. All men 55–74 years of age with no reported histories of prostate cancer or prostatectomy were eligible. Men who had used finasteride in the prior 6 months were not eligible. Half of recruited male participants were randomized to the intervention arm, which included annual prostate-specific antigen (PSA) testing for 5 years and digital rectal examinations (DREs) for 3 years after baseline, and half were randomized to the control arm, which consisted of routine medical care.

At baseline, participants in both arms of PLCO completed a baseline questionnaire, which included questions on weight history, height, and BPH/LUTS. Participants in the intervention arm also completed a food frequency and physical activity questionnaire. In 2004–2008, participants completed a supplemental questionnaire, which included additional questions about BPH/LUTS. Finally, from baseline until active data collection ended in 2015, participants completed brief annual questionnaires to update their cancer information and provide information on finasteride use.

We restricted our analysis to men in the intervention arm (n = 38,340), as only these men provided complete baseline information on physical activity and diet, and underwent annual PSA tests and DREs. We also excluded participants with a history of cancer or evidence of BPH-related outcomes at baseline, missing baseline data, or who did not complete the supplemental questionnaire. Detailed exclusion information is available in the Additional file 1. These exclusion criteria resulted in an incident cohort of 4710 men. Because of the large number of excluded men, we also performed sensitivity analyses excluding only men with the BPH-related outcome of interest from the analysis (e.g., excluding only men with nocturia at baseline from the incident nocturia analysis). These analyses resulted in similar findings as the main analysis.

### Weight and physical activity assessment

On the baseline questionnaire, participants reported their weight in pounds at ages 20, 50, and currently (mean age at baseline = 60.7 years), and their height in feet and inches. We used these values to calculate BMI at ages 20, 50, and baseline, and weight change from ages 20 and 50 to baseline. BMI was categorized as underweight (< 18.5), normal (18.5–24.9), overweight (25–29.9), or obese (≥ 30). Because of small numbers, underweight and normal were combined for analysis. Weight change was categorized as weight loss (> 5 lb), weight maintenance (5 lb loss to 4 lb gain), and increments of weight gain depending on the range of weight gain: 5–14, 15–24, 25–34, 35–44, 45–54, 55–64, 65–74, and ≥ 75 lbs. Participants reported their current levels of “vigorous activities, such as swimming, brisk walking, etc.” and categories of hours per week (none, < 1, 1, 2, 3, and 4 + h/wk) on the baseline questionnaire. We dichotomized physical activity as ≥ 1 versus < 1 h per week.

### BPH-related outcomes and nocturia

Methods used to define BPH-related outcomes are described in detail elsewhere [29, 31]. Briefly, the presence of BPH-related outcomes at baseline was determined using three items from the baseline questionnaire (waking during the night to urinate during the past year [nocturia]; a history of surgical procedures of the prostate, including transurethral resection of the prostate [TURP] and prostatectomy for benign disease; and a history of a physician diagnosis of “an enlarged prostate or benign prostatic hypertrophy”), DRE examiner-estimated baseline prostate volume ≥ 30 cc, and baseline PSA > 1.4 ng/mL [32–35]. Additional details about baseline outcome definitions are noted in Table 1.

**Table 1:** Definitions of incident benign prostatic hyperplasia (BPH)-related outcomes and nocturia

	Nocturia	Physician diagnosis	Finasteride use	Prostate volume ≥ 30 cc	PSA elevation
*Baseline questionnaire*					
Wake 2 + times/night to urinate during a typical night in the past year? (Nocturia)	×	×	×	×	×
Ever been told by a doctor that they have an enlarged prostate or BPH?	×	×	×	×	×
Ever had TURP or prostatectomy for benign disease?	×	×	×	×	×
*Baseline examination*					
Prostate volume^a^ ≥ 30 cc	×	×	×	×	×
PSA > 1.4 ng/mL and no known cancer	×	×	×	×	×
*Supplemental questionnaire*					
Wake 2 + times/night to urinate during a typical night in the past year? (Nocturia)	*				
Ever been told by a doctor that they have an enlarged prostate or BPH?		*			
*Annual study update*					
Taken Proscar or Propecia (Finasteride) in the past year?	×		*	×	×
*Follow-up examination*					
Prostate volume^a^ ≥ 30 cc on any of three DREs				*	
PSA > 1.4 ng/mL on any one of five PSA tests					*
Total	1515	1446	881	2499	1322

× = exclusion criteria; * = inclusion criteria; DRE = digital rectal examination; PSA = prostate-specific antigen

^a^Calculated as (π/6) × width^2^ × length, where width is the transverse measurement and length is the sagittal measurement. Both measurements were estimated by palpation by the trained DRE examiner

Incident BPH-related outcomes and nocturia were defined using data from the supplemental questionnaire, the annual study update questionnaires, and follow-up examinations (Table 1). Two sets of BPH-related questions were included on the supplemental questionnaire: average frequency of waking during the night to urinate in the past year and a physician diagnosis of an enlarged prostate or BPH. We used this information to define incident nocturia [32] and physician diagnosis of an enlarged prostate/BPH. Data from the annual study update questionnaires were used to define incident finasteride use, and data from the follow-up examinations were used to define incident prostate volume ≥ 30 cc), and incident PSA > 1.4 ng/mL.

### Statistical analysis

We investigated associations for body size with incident BPH-related outcomes and nocturia by calculating relative risks (RRs) using Poisson regression with robust variance estimation. All regression models included age. In addition, models for incident self-reported outcomes included time between the dates of completion of the baseline and supplemental questionnaires; those for incident prostate volume ≥ 30 cc included number of follow-up DREs and time between participants’ first and last DRE; and those for incident PSA elevation included number of follow-up PSA tests and time between participants’ first and last PSA test. We investigated as potential confounders factors that might be associated with frequency of medical care including demographic factors, chronic medical conditions, physical activity, and suspected risk-factors for BPH-related outcomes including nutritional factors (see Table 2 for a complete list).

**Table 2:** Age-adjusted baseline characteristics of 4710 male participants eligible for the analysis of body mass index and incident benign prostatic hyperplasia (BPH)-related outcomes and nocturia in the intervention arm of the Prostate, Lung, Colorectal, and Ovarian Cancer Screening Trial by baseline body mass index (BMI), 1993–2001

	Baseline body mass index (kg/m^2^)			All participants
	< 25	25–29	≥ 30	
N	1204	2399	1107	4710
Age (mean, years)	61.1	60.8	60.2	60.7
*Race/ethnicity (%)*				
White	88.3	92.2	92.9	91.3
Black	2.0	1.3	3.0	1.9
Asian/Pacific Islander	8.8	5.2	2.2	5.4
Other	0.9	1.3	1.9	1.4
*Education (%)*				
Less than high school degree	3.8	5.4	6.4	5.2
High school graduate	12.3	18.4	21.6	17.6
Some college/post high school training	28.3	32.5	36.2	32.3
College degree or higher	55.6	43.8	35.9	44.9
*Marital status (%)*				
Married or living as married	83.9	86.7	87.6	86.2
Not currently married	16.1	13.3	12.4	13.8
*Current physical activity (h/week, %)*				
None	11.0	12.4	18.3	13.4
One	24.1	29.5	34.4	29.2
Two to three	30.9	33.0	29.7	31.7
Four or more	34.1	25.1	17.7	25.7
*Smoking history (%)*				
Never smoker	34.4	29.1	24.1	29.3
Current cigarette smoker	13.8	11.0	6.9	10.7
Former cigarette smoker	45.3	51.5	60.3	52.0
Cigar or pipe smoker only	6.5	8.3	8.8	8.0
*Current mean intakes of*				
Energy (kcal/day)	2255.0	2320.4	2445.4	2333.0
Carbohydrates (g/day)	292.3	292.8	300.0	294.3
Fat (g/day)	74.4	79.7	87.0	80.0
Poly-unsaturated fatty acid (g/day)	15.2	15.9	17.0	16.0
Protein (g/day)	85.7	90.7	97.9	91.1
Alcohol (g/day)	18.0	17.1	17.1	17.3
Fruit (servings/day)	2.5	2.3	2.3	2.3
Vegetables (servings/day)	3.8	3.7	3.8	3.8
Red meat (g/day)	87.5	102.3	124.3	103.7
*Dietary*				
Alpha-carotene (mcg/day)	1283.8	1280.5	1316.3	1289.8
Beta-carotene equivalents (mcg/day)	5632.7	5566.4	5611.4	5593.9
Lycopene (mcg/day)	11,205.0	11,839.0	12,667.0	11,716.3
*Total (from the diet and supplements)*				
Beta-carotene (mcg/day)	5463.9	5282.4	5276.2	5327.3
Selenium (mcg/day)	117.6	122.1	130.3	122.9
Vitamin A (IU/day)	15,230.0	14,949.0	14,987.0	15,030.0
Vitamin C (mg/day)	441.8	418.8	377.6	415.0
Vitamin E (IU/day)	164.7	153.3	152.5	156.0
Zinc (mg/day)	20.3	20.3	21.0	20.5
Multivitamin use (%)	48.8	42.7	39.5	43.5
*Medical history (%)*				
Hypertension	15.8	26.9	38.1	26.7
Coronary heart disease	6.8	9.4	11.5	9.2
Stroke	1.0	1.0	1.6	1.2
Diabetes	3.7	4.7	8.7	5.4
Arthritis	19.1	23.5	30.8	24.1
Emphysema	2.0	1.8	1.6	1.8
Bronchitis	2.1	2.0	2.0	2.0
Gall bladder stone or inflammation	3.4	5.6	9.0	5.8
Cirrhosis	0.1	0.2	0.2	0.2
Diverticulosis	3.7	4.3	4.1	4.1
Hepatitis	3.5	3.5	3.1	3.4
Colon polyps and polyp syndromes	6.8	6.6	8.1	7.0
Clinical prostatitis	3.7	3.0	3.3	3.2

g/day: grams per day; h/week: hour per week; IU/day: international unit per day; kcal/day: kilocalories per day; kg/m^2^: kilograms per meter^2^; mcg/day: micrograms per day; mg/day: milligrams per day

To investigate the possible influence of body size throughout the life-course on BPH/LUTS risk, we examined associations for (1) early-life (BMI at age 20), (2) mid-life (BMI at age 50), and (3) later-life (baseline BMI) with incident BPH-related outcomes. These analyses were not mutually adjusted for BMI at different ages, because the small number of men who lost weight over time resulted in small cell sizes and unstable models (Additional file 2: Figure S1).

To further examine associations for body size and weight change throughout the life-course, we investigated associations for weight change from (1) age 20 to baseline and (2) age 50 to baseline with incident BPH-related outcomes. Weight change throughout the life-course was examined in two ways. First, we examined the impact of changing BMI categories throughout the life-course (e.g., a man who went from the normal BMI category at age 20 to the overweight category at baseline compared to a man who remained normal weight throughout the life-course). Second, we examined the impact of smaller increments of weight change throughout the life-course (loss > 5, loss or gain of 5, gain 5–14, gain 15–24, gain 25–34, gain 35–44, gain 45–54, gain 55–64, gain 65–74, gain ≥ 75 lbs) from both (1) age 20 to baseline and (2) age 50 to baseline. These models were adjusted for starting BMI at age 20 or 50 depending on the time interval examined (i.e., models examining weight change from age 20 to baseline were adjusted for BMI at age 20). In secondary analyses, we restricted the analysis to men who did not change BMI category over the time period of interest to further examine the influence of weight change independent of BMI category (i.e., smaller amounts of weight change insufficient to cause a change in BMI category).

To examine the joint influence of body size and physical activity, we performed analyses of baseline physical activity (< 1 vs. ≥ 1 h of activity per week [29]) and incident BPH-related outcomes stratified by baseline BMI category.

## Results

Of the 4710 men in the incident analysis, 1204 (25.6%) participants were under/normal weight, 2399 (50.9%) were overweight, and 1107 (23.5%) were obese at baseline (Table 2). Compared to normal weight men, obese men tended to be younger, and were less likely to be Asian/Pacific Islander, have a college degree or higher, be married, engage in physical activity 4 h/week, and be current smokers, although they were more likely to be former smokers. The most common comorbid chronic conditions were hypertension (26.7%), arthritis (24.1%), and coronary heart disease (9.2%). Obese men were more likely to have hypertension, diabetes, arthritis, and gall bladder stone or inflammation as compared to normal weight men.

Considering BMI earlier in life, 3541 (75.2%) participants were normal weight at age 20 and only 102 (2.2%) were obese. At age 50, 1544 (32.8%) participants were normal weight and 724 (15.4%) were obese. Most participants gained weight over the course of their lives (89.6% gained at least 5 lbs since age 20 with a mean weight gain of 35.9 lbs among those who gained more than 5 lbs), and only a small percentage lost weight (4.0% lost at least 5 lbs since age 20 with a mean weight loss of 17.1 lbs among those who lost more than 5 lbs).

Over the course of follow-up (median = 9 years, range: 5–13), 1515 participants developed nocturia, 1446 reported a new physician diagnosis of BPH, and 881 reported finasteride use (Table 1). With respect to outcomes measured at the follow-up examinations, 2499 men developed a prostate volume ≥ 30 cc over a median of 4 years of follow-up (median number of follow-up DREs = 3) and 1322 developed an elevated PSA over a median of 6 years of follow-up (median number of follow-up PSA tests = 5).

### Later-life (baseline) BMI

Baseline BMI was associated with several definitions of incident BPH-related outcomes (Table 3). Men who were obese were 25% more likely to report nocturia (RR: 1.25, 95% CI 1.11–1.40; p-trend_for continuous BMI_ < 0.0001) than normal weight men. Overweight and obese men were also more likely to have a prostate volume ≥ 30 cc (RR_overweight_: 1.13, 95% CI 1.07–1.21; RR_obese_: 1.10, 95% CI 1.02–1.19; p-trend_for continuous BMI_ = 0.017). However, men who were overweight or obese were less likely to have an elevated PSA (RR_overweight_: 0.89, 95% CI 0.80–0.99; RR_obese_: 0.75, 95% CI 0.65–0.85; p-trend_for continuous BMI_ < 0.0001), and no more likely to report a physician diagnosis of an enlarged prostate/BPH than normal weight men. Associations for finasteride use were difficult to interpret because of the small number of men who used finasteride, but they tended to be consistent in direction and magnitude to associations for nocturia and a prostate volume ≥ 30 cc. Adjusting for covariates did not change any of the associations. However, it is important to note that these findings are not independent of BMI in early- or mid-life (i.e., BMI at ages 20 and 50).

**Table 3:** Age-adjusted risk ratios (RRs) and 95% confidence intervals (CIs) of incident benign prostatic hyperplasia (BPH)-related outcomes and nocturia by body mass index (BMI) across the life-course; Prostate, Lung, Colorectal, and Ovarian Cancer Screening Trial

	< 25		25–29		≥ 30		Per 5 kg/m^2^ increase		p-trend^a^
	RR	95% CI	RR	95% CI	RR	95% CI	RR	95% CI	
*Baseline BMI*									
Nocturia^b^	1.00	Referent	1.07	0.96, 1.18	1.25	1.11, 1.40	1.11	1.06, 1.16	< 0.0001
No. of cases	363		763		389				
Physician Diagnosis^b^	1.00	Referent	1.00	0.90, 1.11	0.95	0.84, 1.07	0.97	0.92, 1.02	0.26
No. of cases	377		748		321				
Finasteride^b^	1.00	Referent	1.07	0.71, 1.61	1.44	0.91, 2.27	1.17	0.96, 1.42	0.11
No. of cases	32		67		40				
Prostate volume ≥ 30 cc^c^	1.00	Referent	1.13	1.07, 1.21	1.10	1.02, 1.19	1.04	1.01, 1.07	0.017
No. of cases	604		1351		544				
Elevated PSA^d^	1.00	Referent	0.89	0.80, 0.99	0.75	0.65, 0.85	0.86	0.81, 0.92	< 0.0001
No. of cases	381		682		259				
*BMI at age 20*									
Nocturia^b^	1.00	Referent	1.09	0.99, 1.20	1.20	0.93, 1.55	1.08	1.01, 1.16	0.026
No. of cases	1124		353		38				
Physician diagnosis^b^	1.00	Referent	0.98	0.88, 1.09	0.88	0.64, 1.22	0.98	0.91, 1.05	0.57
No. of cases	1098		320		28				
Finasteride^b^	1.00	Referent	1.18	0.81, 1.71	^e^		1.09	0.86, 1.39	0.49
No. of cases	103		35						
Prostate volume ≥ 30 cc^c^	1.00	Referent	1.05	0.99, 1.11	1.21	1.05, 1.39	1.07	1.03, 1.12	0.0006
No. of cases	1871		566		62				
Elevated PSA^d^	1.00	Referent	0.98	0.88, 1.10	0.70	0.47, 1.04	0.93	0.86, 1.01	0.074
No. of cases	1006		296		20				
*BMI at age 50*									
Nocturia^b^	1.00	Referent	1.07	0.98, 1.18	1.18	1.04, 1.34	1.09	1.03, 1.15	0.0021
No. of cases	480		794		241				
Physician diagnosis^b^	1.00	Referent	0.92	0.84, 1.01	0.92	0.80, 1.05	0.95	0.93, 1.01	0.13
No. of cases	504		730		212				
Finasteride^b^	1.00	Referent	1.07	0.73, 1.57	1.49	0.93, 2.41	1.18	0.96, 1.46	0.12
No. of cases	42		70		27				
Prostate volume ≥ 30 cc^c^	1.00	Referent	1.08	1.02, 1.15	1.11	1.02, 1.20	1.05	1.02, 1.09	0.0048
No. of cases	796		1341		362				
Elevated PSA^d^	1.00	Referent	0.91	0.82, 1.00	0.75	0.64, 0.89	0.85	0.79, 0.90	< 0.0001
No. of cases	476		682		164				

BPH: benign prostatic hyperplasia; CI: Confidence Interval; BMI: body mass index; RR: risk ratio

^a^For continuous BMI

^b^Also adjusted for time between completion of the baseline and supplemental questionnaires

^c^Also adjusted for number of DREs and time between participants’ first and last DRE

^d^Also adjusted for number of PSA tests and time between participants’ first and last PSA test

^e^Too few cases (n = 1) to estimate

### Early- and mid-life BMI

Similar to the results for baseline BMI, BMI at age 20 was associated with an increased risk of BPH-related outcomes (Table 3). Obese men were 20% more likely to develop nocturia (RR = 1.20, 95% CI 0.93–1.55; p-trend = 0.026) and 21% more likely to develop a prostate volume ≥ 30 cc (RR = 1.21 95% CI 1.05–1.39; p-trend = 0.0006). Results for BMI at age 50 were similar to those for BMI at age 20 with significant findings for nocturia (RR_obese_ = 1.18, 95% CI 1.04–1.34; p-trend = 0.0021) and a prostate volume ≥ 30 cc (RR_obese_ = 1.11, 95% CI 1.02–1.20; p-trend = 0.0048).

### BMI across the life-course

As our findings for BMI at specific ages (i.e., age 20, age 50, and baseline) might reflect the fact that men who are overweight or obese as young men tend to remain overweight or obese throughout life, we next sought to determine the independent effects of BMI at different ages on risk of BPH-related outcomes. This is important to determine the necessary timing of future prevention interventions. Compared to men who remained normal weight throughout life, those who were normal weight at age 20 and became overweight by baseline had slightly increased risks of nocturia (RR = 1.09, 95% CI 0.98–1.22) and a prostate volume ≥ 30 cc (RR = 1.12, 95% CI 1.05–1.20) and those who were normal weight at age 20 and became obese had an increased risk of nocturia (RR = 1.28, 95% CI 1.10–1.47, Table 4). Men who were overweight at age 20 who became obese also had an increased risk of nocturia relative to those who remained overweight throughout life (RR = 1.49, 95% CI 1.00–2.21). Generally similar findings were observed for cross-categories of BMI at age 50 and baseline BMI. Together, these findings suggest that later-life overweight and obesity (i.e., at ≥ 55 years of age, the minimum age of entry into PLCO) contributes independently, of early-life body size, to nocturia, and that later-life overweight contributes independently to a prostate volume ≥ 30 cc.

**Table 4:** Age-adjusted risk ratios (RRs) and 95% confidence intervals (CIs) of incident benign prostatic hyperplasia (BPH)-related outcomes and nocturia by body mass index (BMI) change across the life-course; Prostate, Lung, Colorectal, and Ovarian Cancer Screening Trial

		Baseline BMI (kg/m^2^)					
		< 25		25–29		≥ 30	
		RR	95% CI	RR	95% CI	RR	95% CI
**BMI at 20 (kg/m**^**2**^**)**							
< 25	Nocturia^a^	1.00	Referent	1.09	0.98, 1.22	1.28	1.10, 1.47
	No. of cases	336		609		179	
	Prostate volume ≥ 30 cc^b^	1.00	Referent	1.12	1.05, 1.20	1.04	0.94, 1.15
	No. of cases	572		1058		241	
25–29	Nocturia^a^	1.40	1.03, 1.90	1.09	0.93, 1.28	1.25	1.08, 1.45
	No. of cases	26		150		177	
	Prostate volume ≥ 30 cc^b^	0.94	0.73, 1.20	1.16	1.06, 1.26	1.10	1.00, 1.21
	No. of cases	31		280		255	
≥ 30	Nocturia^a^	^c^		^c^		1.41	1.07, 1.86
	No. of cases					33	
	Prostate volume ≥ 30 cc^b^	^c^		1.61	1.32, 1.97	1.25	1.05, 1.48
	No. of cases			13		48	
**BMI at 50 (kg/m**^**2**^**)**							
< 25	Nocturia^a^	1.00	Referent	1.09	0.93, 1.28	^c^	
	No. of cases	317		158			
	Prostate volume ≥ 30 cc^b^	1.00	Referent	1.07	0.98, 1.18	^c^	
	No. of cases	539		253			
25–29	Nocturia^a^	1.04	0.80, 1.34	1.06	0.94, 1.19	1.30	1.13, 1.49
	No. of cases	44		561		189	
	Prostate volume ≥ 30 cc^b^	0.87	0.72, 1.05	1.13	1.06, 1.21	1.06	0.96, 1.17
	No. of cases	60		1029		252	
≥ 30	Nocturia^a^	^c^		1.23	0.96, 1.58	1.21	1.05, 1.41
	No. of cases			44		195	
	Prostate volume ≥ 30 cc^b^	^c^		1.16	1.00, 1.36	1.10	1.00, 1.21
	No. of cases			69		288	

BPH: benign prostatic hyperplasia; CI: Confidence Interval; BMI: body mass index; RR: risk ratio

^a^Also adjusted for time between completion of the baseline and supplemental questionnaires

^b^Also adjusted for number of DREs and time between participants’ first and last DRE

^c^Too few cases (n ≤ 5) to estimate

Too few men were overweight or obese in early-life and too few men lost large amounts of weight through the life-course to investigate the independent effects of early-life overweight and obesity with confidence.

### Incremental and within BMI category weight change across the life-course

Findings for weight change from ages 20 and 50 (Table 5) were consistent with those from the BMI cross-category analyses. For weight change from age 20 to baseline, risk of nocturia increased slightly for a 35–44 lb weight gain and increased to a greater degree for a ≥ 45 lb gain; smaller amounts of weight gain, particularly those insufficient to cause an increase in BMI category, were not associated with increased nocturia risk. Weight gain sufficient to cause a change in BMI category was also necessary to increase risk of a prostate volume ≥ 30 cc. Similar findings were observed for weight change from age 50 to baseline as for weight change from age 20 to baseline. Weight loss did not appear to be protective for either nocturia or a prostate volume ≥ 30 cc, but the amount of weight loss in the cohort was small.

**Table 5:** Age-adjusted risk ratios (RRs) and 95% confidence intervals (CIs) of incident benign prostatic hyperplasia (BPH)-related outcomes and nocturia by weight change across the life-course; Prostate, Lung, Colorectal, and Ovarian Cancer Screening Trial

	Loss > 5		Loss or gain of 5		Gain 5–14		Gain 15–24		Gain 25–34		Gain 35–44		Gain 45–54		Gain 55–64		Gain 65–74		Gain ≥ 75		p-trend^a^
	RR	95% CI	RR	95% CI	RR	95% CI	RR	95% CI	RR	95% CI	RR	95% CI	RR	95% CI	RR	95% CI	RR	95% CI	RR	95% CI	
**Weight change from age 20 to baseline (lbs)**																					
*All participants*																					
Nocturia^a^	1.15	0.89, 1.48	1.00	Referent	0.96	0.78, 1.18	1.00	0.82, 1.21	1.05	0.87, 1.28	1.08	0.89, 1.32	1.13	0.92, 1.39	1.33	1.07, 1.64	1.16	0.90, 1.49	1.21	0.96, 1.52	0.0028
No. of cases	68		93		183		241		254		219		169		128		67		93		
Prostate volume ≥ 30 cc ^b^	1.09	0.93, 1.28	1.00	Referent	1.11	0.98, 1.25	1.10	0.97, 1.24	1.08	0.95, 1.21	1.14	1.00, 1.28	1.16	1.02, 1.32	1.10	0.96, 1.27	1.13	0.96, 1.32	1.11	0.95, 1.29	0.18
No. of cases	106		155		349		431		428		363		273		170		102		122		
*Restricted to those who did not change body mass index category from age 20 to baseline*																					
Nocturia^a^	1.09	0.79, 1.50	1.00	Referent	0.93	0.74, 1.16	1.04	0.83, 1.31	0.94	0.70, 1.25	0.90^a^	0.64, 1.25									0.97
No. of cases	40		88		134		137		69		51										
Prostate volume ≥ 30 cc ^b^	1.10	0.90, 1.33	1.00	Referent	1.06	0.92, 1.21	1.07	0.93, 1.23	1.02	0.85, 1.22	0.92^a^	0.75–1.13									0.76
No. of cases	66		147		258		227		121		81										
**Weight change from age 50 to baseline (lbs)**																					
*All participants*																					
Nocturia ^a^	1.10	0.89, 1.35	1.00	Referent	0.99	0.89, 1.11	1.17	0.96, 1.42	1.23	0.90, 1.68	1.38 ^a^	1.01, 1.90									0.0039
No. of cases	194		434		456		242		97		92										
Prostate volume ≥ 30 cc ^b^	1.03	0.91, 1.16	1.00	Referent	1.02	0.95, 1.08	1.04	0.92, 1.16	0.93	0.77, 1.14	1.14 ^a^	0.83, 1.01									0.32
No. of cases	310		777		807		373		125		107										
*Restricted to those who did not change body mass index category from age 50 to baseline*																					
Nocturia^a^	1.06	0.78, 1.44	1.00	Referent	1.07	0.90, 1.27	1.07^a^	0.80, 1.42													0.28
No. of cases	85		242		155		37														
Prostate volume ≥ 30 cc ^b^	0.99	0.83, 1.19	1.00	Referent	1.03	0.93, 1.14	1.03^a^	0.87, 1.23													0.25
No. of cases	146		436		263		55														

^a^Includes values of weight gain greater than the upper bound of the weight category

### Physical activity and BMI

Physical activity was not associated with nocturia risk among obese men (RR = 0.94, 95% CI 0.80–1.10), but was associated with a reduced risk of nocturia among overweight men (RR = 0.85, 95% CI 0.75–0.96). Normal weight men had a non-significant risk reduction similar to that of overweight men (RR = 0.86, 95% CI 0.71–1.04). No associations were observed for physical activity and a prostate volume ≥ 30 cc in normal weight, overweight, or obese men (RRs = 0.99–1.04).

## Discussion

This report expands on previous research on body size and BPH by examining body size in relation to *incident*, as opposed to *prevalent*, BPH-related outcomes and nocturia, and by examining body size across the life-course as opposed to later in life only. Our findings suggest that greater later-life body size (overweight or obesity) is associated with adverse BPH-related outcomes, independent of early-life body size. Specifically, becoming overweight or obese, but not gaining smaller amounts of weight throughout the life-course, was associated with increased risks of nocturia and a prostate volume ≥ 30 cc. This is important as it suggests that interventions to reduce overweight or obesity even later in life have the potential to prevent adverse BPH-related outcomes. Too few men were obese early in life or lost large amounts of weight to determine the independent effects of greater early-life body size. While rare in PLCO, early-life obesity will become important to understand with the growing obesity epidemic. Finally, although we found that engaging in physical activity reduced the risk of nocturia for normal and overweight men, it did not reduce the risk for obese men or the risk of a prostate volume ≥ 30 cc for men of any weight, suggesting the need for other prevention strategies.

### Later-life body size

Our results for later-life body size are generally consistent with the literature to date, including two meta-analyses [8, 9]. Although a strength of our study is that we specifically examined incident BPH, our findings were generally consistent with those that examined prevalent BPH. Our positive findings for nocturia are similar to those from most existing studies of body size and incident BPH or LUTS (or composite endpoints including LUTS, which likely dominated these endpoints) [8, 15, 17, 20, 21, 36]. Our findings for a prostate volume ≥ 30 cc are also similar to those from most previous studies of prevalent large prostate volume/weight, as well as to those that examined prostate growth over time. [8, 9, 11, 13, 36–42]. In addition, although our findings for finasteride use were not statistically significant, they are still consistent in direction and magnitude to those from most previous studies of incident BPH/LUTS and prevalent prostate volume. Finally, our protective findings for overweight/ obesity and an elevated PSA are similar to those from many previous studies [43–46], and likely reflect the influence of overweight/obesity on blood volume rather than on prostate size.

Despite the similarity of our findings to those from many previous studies, they do still differ from a handful of previous studies [14, 16, 19, 23, 47]. These differences could be partly attributed to differences in patient populations (e.g., Asian versus North American populations); variations in ages of BMI ascertainment (e.g., mid- versus late-life), inconsistencies in both exposure and outcome definitions (e.g., continuous versus categories of BMI, varying composite definitions of BPH, outcomes ascertained by ICD-9 codes versus self-report, and changes in clinical practice over the decades captured by these studies). In addition, our null results for a physician diagnosis of BPH differ from our positive or suggestively positive findings for nocturia and finasteride use. This difference may possibly be explained by the more stringent criteria required for a physician diagnosis rather than self-report of LUTS.

### Body size through the life-course

Despite growing interest in a life-course approach to disease causation, few studies have examined how early-life factors or changes in risk factors may alter risk of BPH-related outcomes. This information is critical for chronic conditions as it provides key information on when interventions should be targeted. In our analyses, we found a clear association between gaining large amounts of weight (at least 45 lbs) and risk of nocturia, similar to findings from one [17], but not another [23] study of weight change and incident BPH-related outcomes. Specifically, in the study by Mondul et al., weight gain from age 21 was significantly associated with risk of moderate or worse LUTS only among men who gained at least 40 lbs, and with progression to severe LUTS among those who gained at least 30 lbs [17]. By contrast, Gupta et al., did not observe an association between weight change per decade (mean weight change per decade 4–5 kg) and the development of BPH [23]. Together, these findings suggest that a sizeable weight gain of at least 30–40 lbs throughout the life-course is needed before the risk for LUTS increases.

Despite the large sample size of PLCO, one limitation in this analysis is that too few men were obese early in life and too few lost weight to inform: (1) the independent contribution of early-life body size on the risks of nocturia and a large prostate, and (2) weight loss as a possible prevention strategy for BPH/LUTS. Cohorts with greater prevalences of early-life obesity, such as more contemporary cohorts, and cohorts with greater prevalences of weight loss will be required to answer these questions. All that we can say with a greater degree of certainty from our analyses is that being overweight or obese as an older man was associated with increased risks of developing nocturia and a prostate volume ≥ 30 cc.

### Physical activity

Because previous studies have indicated a role for physical activity in BPH/LUTS [29], we examined the joint association of activity and obesity. Our findings suggest that the physical activity benefit may be limited to normal and overweight men, and that among obese men, the effects of obesity trump any potential benefit of physical activity. This finding, which is consistent with that observed in the Southern Community Study [20], is unfortunate because other management strategies and therapies, such as finasteride, have also had limited efficacy in obese men [40, 48].

Our study results must be interpreted in light of some limitations. First, it is difficult to accurately measure prostate volume, particularly in obese men [43]. This could have contributed to our weaker dose–response for increasing BMI with risk of DRE-estimated prostate volume ≥ 30 cc than for nocturia. In addition, weight was based on self-report and men were asked to recall their weight decades earlier. However, despite this, we were still able to observe significant associations between both early- and mid-life BMI and BPH/LUTS, as well as between weight gain and BPH/LUTS. Previous research has shown that middle-aged men can accurately recall both height and weight 27–37 years later, and, on average, weight gain is only underestimated by 3 kg and BMI by 1 kg/m^2^ [49]. It is also important to note that BMI may be a less accurate measure of obesity in younger, muscular men, as it does not distinguish between muscle mass and body fat [50]. Moreover, unmeasured components of medical history, including use of certain medications such as diuretics, could have biased our results. Unmeasured medical conditions could have also impacted our findings, however, our assessment of common comorbidities (see Table 2 for full list) including diabetes, indicated that they were not significant confounders. Finally, our measure of BPH-related outcomes only included one LUTS–nocturia. Thus, men with LUTS other than nocturia, particularly milder storage LUTS (e.g., urinary urgency and frequency) that would be unlikely to contribute to a physician diagnosis of BPH or finasteride use, may not have been captured by our BPH-related outcome definitions.

Despite these limitations, our study has several strengths. These include ascertaining incident rather than prevalent BPH/LUTS, and measuring BMI at multiple-time points including early-, mid-, and later-life.

## Conclusion

We found that obesity was associated with incident nocturia and a prostate volume ≥ 30 cc. Importantly, we were able to show that late-life BMI was associated with risk of BPH/LUTS independent of early-life BMI, indicating that interventions later in life have the potential to reduce BPH/LUTS. Our results further suggest that nocturia may be prevented or reduced through weight management, but that exercise alone may not be as effective in reducing nocturia risk in obese men.

## Supplementary Information

**Additional file 1.** Study exclusion criteria.

**Additional file 2: Supporting Figure 1.** Average weight change in pounds from ages 20 and 50 to baseline, by BMI Category.

## Data Availability

PLCO data is available to the general scientific community: https://cdas.cancer.gov/learn/plco/instructions/?type=data.

## Abbreviations

BPH: Benign Prostatic Hyperplasia
PLCO: Prostate, Lung, Colorectal, and Ovarian Cancer Screening Trial
RR: Relative Risk
CI: Confidence Interval
LUTS: Lower Urinary Tract Symptoms
DRE: Digital Rectal Exam
TURP: Transurethral Resection of the Prostate
PSA: Prostate Specific Antigen
BMI: Body Mass Index
