# Supplementary material for: Body size throughout the life-course and incident benign prostatic hyperplasia-related outcomes and nocturia
Source: BMC Urol. 2021 Mar 27;21:47. doi: 10.1186/s12894-021-00816-5 (PMC8005244; doi:10.1186/s12894-021-00816-5)
Supplement: Supplementary file 1 — Additional file 1. Study exclusion criteria. [file 12894_2021_816_MOESM1_ESM.docx]

**Body size throughout the life-course and incident benign prostatic hyperplasia-related outcomes and nocturia**

Khan S, Wolin KY, Pakpahan R, Grubb III RL, Colditz GA, Ragard L, Mabie J, Breyer BN, Andriole GL, and Sutcliffe S.

**STUDY EXCLUSION CRITERIA**

We excluded participants who: a) reported a history of cancer (except basal or squamous-cell skin cancer) at baseline (n=827); b) were diagnosed with prostate cancer on the baseline prostate cancer screen (n=609); c) did not complete the baseline questionnaire (n=887); d) provided incomplete information on BPH-related outcomes (n=64); e) missed or had an invalid baseline PSA test or DRE result (only among those who did not report BPH surgery, n=3,023); f) did not complete or provided incomplete data for the baseline food frequency and physical activity questionnaire (3,997); and g) were missing BMI or physical activity information (n=539). To investigate incident BPH-related outcomes and nocturia, we further excluded men who: a) had any evidence of BPH-related outcomes or nocturia at baseline (described below, n=21,927); b) were diagnosed with prostate cancer before completion of the supplemental questionnaire (n=101); c) did not complete the supplemental questionnaire (n=1,556); d) provided incomplete information on BPH/LUTS on the supplemental questionnaire (n=84); and e) did not have either a valid follow-up PSA test or DRE result (only among those who did not report finasteride use, n=26).
