# Supplementary material for: Body size throughout the life-course and incident benign prostatic hyperplasia-related outcomes and nocturia
Source: BMC Urol. 2021 Mar 27;21:47. doi: 10.1186/s12894-021-00816-5 (PMC8005244; doi:10.1186/s12894-021-00816-5)
Supplement: Supplementary file 2 — Additional file 2: Supporting Figure 1. Average weight change in pounds from ages 20 and 50 to baseline, by BMI Category. [file 12894_2021_816_MOESM2_ESM.docx]

**Body size throughout the life-course and incident benign prostatic hyperplasia-related outcomes and nocturia**

Khan S, Wolin KY, Pakpahan R, Grubb III RL, Colditz GA c, Ragard L, Mabie J, Breyer BN, Andriole GL, and Sutcliffe S.


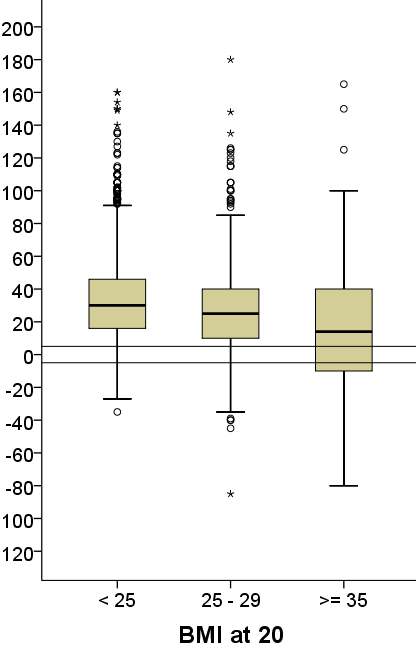

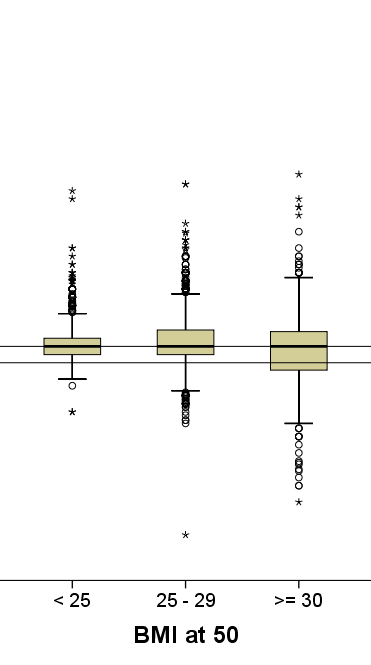

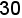

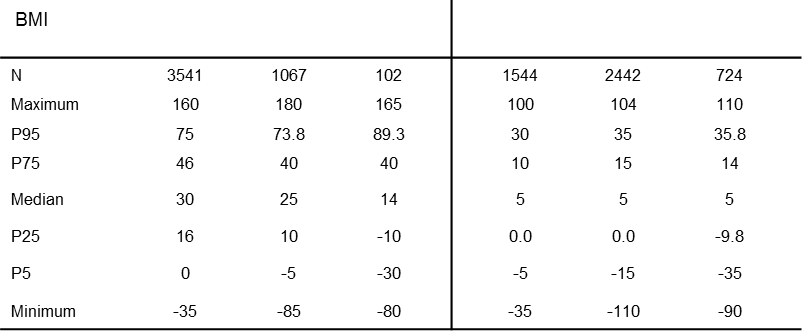


**Weight Change**

**BMI at age 20**

**BMI at age 50**

**Additional file 2: Figure S1.** Average weight change in pounds from ages 20 and 50 to baseline, by BMI Category
